# Lost in traffic: consequences of altered palmitoylation in neurodegeneration

**DOI:** 10.3389/fphys.2023.1166125

**Published:** 2023-05-30

**Authors:** Firyal Ramzan, Fatima Abrar, Gyana Gourab Mishra, Lucia Meng Qi Liao, Dale D. O. Martin

**Affiliations:** NeurdyPhagy Lab, Department of Biology, Faculty of Science, University of Waterloo, Waterloo, ON, Canada

**Keywords:** palmitoylation, neurodegenenerative diseases, autophagy, posttranslational modification, sex differences

## Abstract

One of the first molecular events in neurodegenerative diseases, regardless of etiology, is protein mislocalization. Protein mislocalization in neurons is often linked to proteostasis deficiencies leading to the build-up of misfolded proteins and/or organelles that contributes to cellular toxicity and cell death. By understanding how proteins mislocalize in neurons, we can develop novel therapeutics that target the earliest stages of neurodegeneration. A critical mechanism regulating protein localization and proteostasis in neurons is the protein-lipid modification S-acylation, the reversible addition of fatty acids to cysteine residues. S-acylation is more commonly referred to as S-palmitoylation or simply palmitoylation, which is the addition of the 16-carbon fatty acid palmitate to proteins. Like phosphorylation, palmitoylation is highly dynamic and tightly regulated by writers (i.e., palmitoyl acyltransferases) and erasers (i.e., depalmitoylating enzymes). The hydrophobic fatty acid anchors proteins to membranes; thus, the reversibility allows proteins to be re-directed to and from membranes based on local signaling factors. This is particularly important in the nervous system, where axons (output projections) can be meters long. Any disturbance in protein trafficking can have dire consequences. Indeed, many proteins involved in neurodegenerative diseases are palmitoylated, and many more have been identified in palmitoyl-proteomic studies. It follows that palmitoyl acyl transferase enzymes have also been implicated in numerous diseases. In addition, palmitoylation can work in concert with cellular mechanisms, like autophagy, to affect cell health and protein modifications, such as acetylation, nitrosylation, and ubiquitination, to affect protein function and turnover. Limited studies have further revealed a sexually dimorphic pattern of protein palmitoylation. Therefore, palmitoylation can have wide-reaching consequences in neurodegenerative diseases.

## 1 Introduction

Regardless of the etiology, one of the first insults in all neurodegenerative diseases is mislocalization of proteins. Protein mislocalization in neurons is often linked to proteostasis deficiencies, which can lead to a build-up of misfolded proteins or damaged organelles, ultimately resulting in cell death (Martin et al., 2015; Suk and Rousseaux, 2020). In this review, we discuss the contribution of palmitoylation to protein dysfunction related to neurodegeneration. S-palmitoylation, the reversible addition of the lipid palmitate to specific cysteine residues via a labile thioester bond, helps to anchor proteins to membranes, allowing dynamic regulation of protein localization. Thus, the reversibility of S-palmitoylation allows soluble proteins to be directed to and from membranes throughout neurons, including synapses, dendrites, and axons. Palmitoylation can also occur on transmembrane proteins, dynamically regulating their function without affecting localization (Zaballa and van der Goot, 2018). Irreversible palmitoylation is less common but possible via a stable amide bond (N-palmitoylation) or ester bond (O-palmitoylation) (Buglino and Resh, 2008; Ji et al., 2016; Gao and Hannoush, 2018). In this review, however, palmitoylation refers to S-palmitoylation.

**FIGURE 1:**
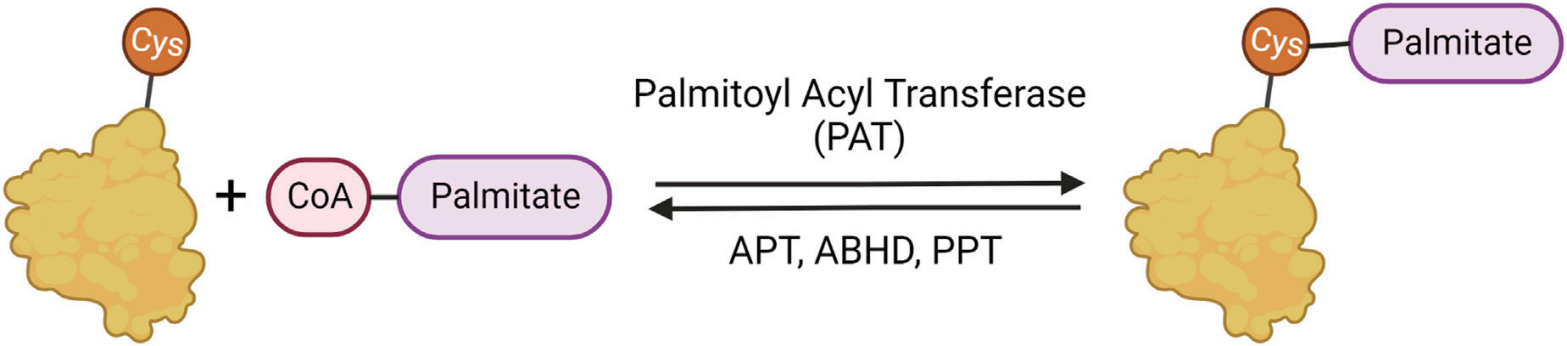
Palmitoylation mechanism. Palmitoylation involves the reversible addition of palmitate to cysteine residues through a labile thioester bond. The palmitate is added to proteins by palmitoylate acyl transferases (PATs) and removed by α/β serine hydrolases (ABHDs) and thioesterases (APTs and PPTs).

Palmitoylation is important in many cellular and metabolic processes and has been implicated in neurodegeneration and diseases of the nervous system (Resh, 1999; 2016; Linder and Deschenes, 2007; Sanders et al., 2015; Cho and Park, 2016) The addition of the 16-carbon fatty acid palmitate to cysteine residues is facilitated by palmitoyl acyl transferases (PATs) that are characterized by and named after their conserved Asp-His-His-Cys with Zinc fingers (ZDHHC) active site domains (Fukata et al., 2004; Roth et al., 2006; Hou et al., 2009). The removal of palmitate from proteins is facilitated by several classes of serine hydrolases (Figure 1), including acyl protein thioesterase (APTs), protein palmitoyl thioesterase (PPTs), and α/β-hydrolase domain (ABHD) proteins (Chen, Fan, and Boehning, 2021; Bononi et al., 2021; Hornemann, 2015; Lin and Conibear, 2015; Toyoda, Sugimoto, and Yamashita, 1999; Duncan and Gilman, 1998; Yokoi et al., 2016).

## 2 Going off course: dire consequences of altered palmitoylation in neurons

Palmitoylation regulates many aspects of neuronal protein trafficking and function (reviewed in Philippe and Jenkins, 2019; Petropavlovskiy et al., 2021). Neurons are polarized cells with long thin axons and shorter, thicker dendrites. Using action potentials that run from the cell body down the length of the axon, neurons communicate with each other through synapses, the connection points between axons and dendrites from different neurons (Bullock et al., 2005). At the synapse, distinct proteins and receptors localize to the post-synapse and pre-synapse. A recent compendium of 15 palmitoyl-proteome studies, found an enrichment of palmitoylation in the synaptic proteome (Sanders et al., 2015). Now, nearly 50% of synaptic proteins are considered to be palmitoylated (Petropavlovskiy et al., 2021). The reversibility of palmitoylation allows neurons to dynamically create and maintain this specific protein localization (Matt et al., 2019; Philippe and Jenkins, 2019). Thus, the quick reversibility of palmitoylation and its importance in regulating protein localization makes it a compelling candidate for functional regulation in neurodegenerative diseases.

Not surprisingly, a mammalian palmitoyl-proteome study identified numerous biomarker-based disease-association enrichments of neurodegenerative diseases and neurodevelopmental disorders (Sanders et al., 2015). These diseases and disorders include, but are not limited to, schizophrenia, chorea, Huntington Disease (HD), spinal cord diseases, Amyotrophic Lateral Sclerosis (ALS), and cranial nerve disease (Sanders et al., 2015). Indeed, key proteins involved in HD, ALS, and Alzheimer Disease (AD) have already been shown to have altered palmitoylation in disease states (Yanai et al., 2006; Cho and Park, 2016; Antinone et al., 2017).

Considering the enrichment of palmitoylated proteins and the dynamic role it plays in cells, palmitoylation has emerged as an attractive area of research in neurodegenerative diseases. The role of palmitoylating and depalmitoylating enzymes in neurodegenerative diseases is discussed in the next section. Importantly, palmitoylation acts as a mediator and modulator of numerous functions within the cell, but is best known for shuttling soluble proteins to membranes. However, palmitoylation does not act alone, but in concert with or in addition to other posttranslational modifications, discussed in section 4. The interplay of palmitoylation with other posttranslational modifications is tightly regulated and important for proteostasis, which is disrupted in numerous diseases (See section 5). Finally, there is growing evidence of sex differences in palmitoylation, indicating a role of palmitoylation in the regulation of sex differences in disease, discussed in section 6.

## 3 Putting together the ancestral puzzle of palmitoylation: Lessons from model organisms

Studying protein palmitoylation using lower model organisms like *Saccharomyces cerevisiae*, *Caenorhabditis elegans*, *Drosophila melanogaster* and *Mus musculus* has immense potential to provide critical insights due to the conserved role of palmitoylation and associated pathological processes contributing to neurodegeneration (Hayashi, 2021). Studies have identified 7 PATs in *Saccharomyces cerevisiae*, 15 in *Caenorhabditis elegans*, 22 in *Drosophila melanogaster* and 24 in *Mus musculus* (Bannan et al., 2008; Edmonds and Morgan, 2014; Zaballa and van der Goot, 2018). All these PATs share the characteristic cysteine-rich domain (CRD) containing the “DHHC” motif unique to palmitoylating enzymes highlighting the conserved mechanisms PATs use in palmitoylating their substrates. Importantly, model organisms have and continue to serve as a valuable tool to decipher the role of palmitoylation in cellular function and pathogenesis by facilitating the ability of researchers to run a diverse set of genetic manipulation, localization studies, and behavioural assays.

### 3.1 Model organisms used in palmitoylation studies

Yeast was the first model system used to identify palmitoylation through the identification of zinc-finger-like CRD containing palmitoylating enzymes Akr1 and Erf2 (Lobo et al., 2002; Roth et al., 2002) that eventually led the way to the identification of a family of 23–24 ZDHHC isoforms in mammals (Fukata et al., 2004; Huang et al., 2004; Keller et al., 2004). Yeast has been an invaluable model organism to identify PAT interactors through high throughput knock-out studies, which has helped identify palmitoylation of various neurodegeneration proteins. The fruit fly model, *Drosophila melanogaster*, is emerging as another model organism for studying palmitoylation and has been key in modelling neurodegenerative diseases. Flies allow sophisticated genetic manipulation and molecular analysis at an organismal level that can be coupled with various behavioural assays (Lu and Vogel, 2009; Nitta and Sugie, 2022).

### 3.2 Uses of model organisms in high throughput studies

Apart from PAT substrate identification, yeast has provided a robust and efficient model for high throughput screening of the global S-palmitoyl-proteome under various nutrient conditions (Roth et al., 2006) as well as identification of palmitoylation inhibitors (Coronel Arrechea et al., 2021). Once validated, these inhibitors could be translated to higher model organisms and ultimately lead to palmitoylation-related therapeutic development for neurodegenerative diseases. While yeast has been an invaluable model to identify PATs, PAT substrates, establish high throughput approaches, and identify pharmacological inhibitors, they are unable to provide understanding of the nervous system. To inform the role of palmitoylation in the nervous system and organisms at a global scale, *Drosophila melanogaster* and *Mus musculus* have been used.

The Korey group (Bannan et al., 2008) was the first to identify all 22 PATs and their expression profile in flies. Many fly PATs are highly expressed in the nervous system, emphasizing palmitoylation’s role in neuronal health and function. A recent S-palmitoyl-proteome study in *Drosophila*-derived S2R+ cells identified an estimated 3.5% of expressed genes in these cells to be palmitoylated. Interestingly, these palmitoylated proteins were found to be more conserved between flies and mammals compared to non-palmitoylated proteins (Porcellato et al., 2022). They also identified putative substrates and interaction partners of the various PATs through DHHC-BioID, a proximity biotinylation-based method, which they validated in larval and adult flies for a complete organismal study. Although the function of each PAT in fly neuronal health and their conservation to the mammalian system remains to be characterized, this work provides the foundation for more in-depth studies involving PATs in the nervous system and their roles in neurodegeneration.

The utilization of mice in recent RNA sequencing studies has resulted in the curation of a valuable resource for palmitoylation enzymes in the mouse brain, BrainPalmSeq (Wild et al., 2022). This resource combines information from numerous single-cell RNAseq studies, complemented with bulk and pooled-cell RNAseq studies which include a diversity of brain regions, and ages of tested mice. The authors revealed cell-type-specific and regional ZDHHC expression in the nervous system. They further found interrelated patterns of expression between ZDHHC enzymes and their substrates, as well as depalmitoylating enzymes and other brain-expressed genes (Wild et al., 2022). They recently expanded on this to create CellPalmSeq, a new interactive tool and RNAseq database for multiple human and laboratory cell lines (Wild et al., 2023). The same group identified substrates that are differentially palmitoylated and speculated based on co-localization analysis that the dynamic palmitoylation of a subset of these substrates was mediated by ZDHHCs 2, 5, and 8 (Nasseri et al., 2022) This suggests that palmitoylation in the brain is highly regulated and influenced by external stimuli. Thus, this information provides valuable insight into the roles of palmitoylating enzymes in the mammalian brain and nervous system.

### 3.3 Putting PATs in their place: how PATs fit in neurodegeneration

In this section, we outline the roles and contributions of specific PATs in mechanisms of neurodegeneration and diseases of the brain and nervous system as known in the current literature.

#### 3.3.1 Huntington disease (ZDHHC17 and ZDHHC13)

Huntington Disease (HD) is an autosomal-dominant progressive neurodegenerative disease caused by a mutation in the *HTT* gene. This single mutation results in an expansion in the polyglutamine (polyQ or CAG repeat in the *HTT* gene) region at the N-terminus of the huntingtin (HTT) protein (The Huntington’s Disease Collaborative Research Group, 1993). Repeats greater than 36 result in mutant huntingtin (mHTT) and increases the risk of developing HD. The larger the repeat region, the earlier the age of onset and the more severe the symptoms. In HD, mHTT has reduced palmitoylation compared to the wild-type form, which is linked to increased insoluble mHTT and decreased ZDHHC17 activity (Yanai et al., 2006; Huang et al., 2011). Increasing brain palmitoylation in HD mouse models acts to rescue the phenotype and is emerging as a potential therapeutic approach (Lemarié et al., 2021; Virlogeux et al., 2021).

ZDHHC17 is the most well-conserved PAT, from yeast to humans (Young et al., 2012; Butland et al., 2014). Both ZDHHC17 and ZDHHC13 are unique among the PATS due to the presence of their N-terminal ankyrin-repeat domains (facing the cytosol), absent in other PATs (Mitchell et al., 2006; Tabaczar et al., 2017). Moreover, they have 6 predicted transmembrane domains as opposed to the typical 4, also reflected in the yeast ZDHHC17 homolog Akr1 (Politis, Roth, and Davis, 2005; Mitchell et al., 2006; Tabaczar et al., 2017). ZDHHC17 was initially discovered as an interactor protein of HTT *via* a yeast two-hybrid screen (Singaraja et al., 2002) and was initially named Huntingtin Interacting Protein 14 (HIP14). Due to its similarity to ZDHHC17, ZDHHC13 was named HIP14-Like or HIP14 L. The HTT–ZDHHC17 interaction is disrupted by the pathogenic expansion of the mHTT polyQ domain (128Q compared with 15Q for the wild-type protein) (Singaraja et al., 2002). The study was later validated in an HD mouse model, suggesting that defects in HTT palmitoylation could contribute to the disease (Yanai et al., 2006).

ZDHHC17 and ZDHHC13 are essential for regulating mHTT function in HD, specifically through palmitoylation (Huang et al., 2011). Further, in the presence of mHTT with an expanded polyQ tract in the YAC128 HD mouse model, there is reduced HTT palmitoylation and reduced interaction between HTT and ZDHHC17 (Singaraja et al., 2002; Huang et al., 2004; Yanai et al., 2006). In a mouse model expressing truncated ZDHHC13 that retains the N-terminal ankyrin repeat, mice have abnormalities in hair, skin, and bone, as well as reduced survival and increased amyloidosis, indicating a role of ZDHHC13 in regulating essential functions in multiple tissue and cell types, including neurons, epithelial, and bone (Saleem et al., 2010). Interestingly, a less severe phenotype is linked to *Zdhhc13* knockout. This may suggest that the ankyrin repeat has a dominant negative effect in the truncation model (Sutton et al., 2013). In contrast, mouse models of *Zdhhc17* deficiency or deletion have more severe phenotypes. In the constitutive *Zdhhc17* hypomorph model, the resulting behavioural and physiological deficits resemble those of an HD mouse model (Singaraja et al., 2011). In the conditional inducible *Zdhhc17* knockout mouse model, adulthood deletion of *Zdhhc17* resulted in sudden death due to rapidly progressing paralysis within 10 weeks post-induction (Sanders et al., 2016). These data suggest a significant role of ZDHHC17 in modulating pathology in HD, but also suggest that ZDHHC17 is an essential PAT, likely due to its many interactors and substrates (Butland et al., 2014). This may be linked to recently identified ZDHHC17 substrates dual leucine-zipper kinase (DLK) and nicotinamide mononucleotide adenylyltransferase-2 (NMNAT2) (Niu et al., 2020). DLK and NMNAT2 are involved in a compartmentalized retinal ganglion cell death model where these enzymes form a “trust but verify” system. Specifically, palmitoylated DLK is involved in conveying an axon-to-soma pro-degenerative signal in the event of a neuronal insult like axon injury, through upregulation of pro-apoptotic genes, while palmitoylated NMNAT2 is important in maintaining axon integrity through inhibition of Wallerian degeneration. Thus, ZDHHC17-mediated palmitoylation of DLK and NMNAT2 may be a critical regulator that decides neuronal cell fate (Niu et al., 2020).

**FIGURE 2:**
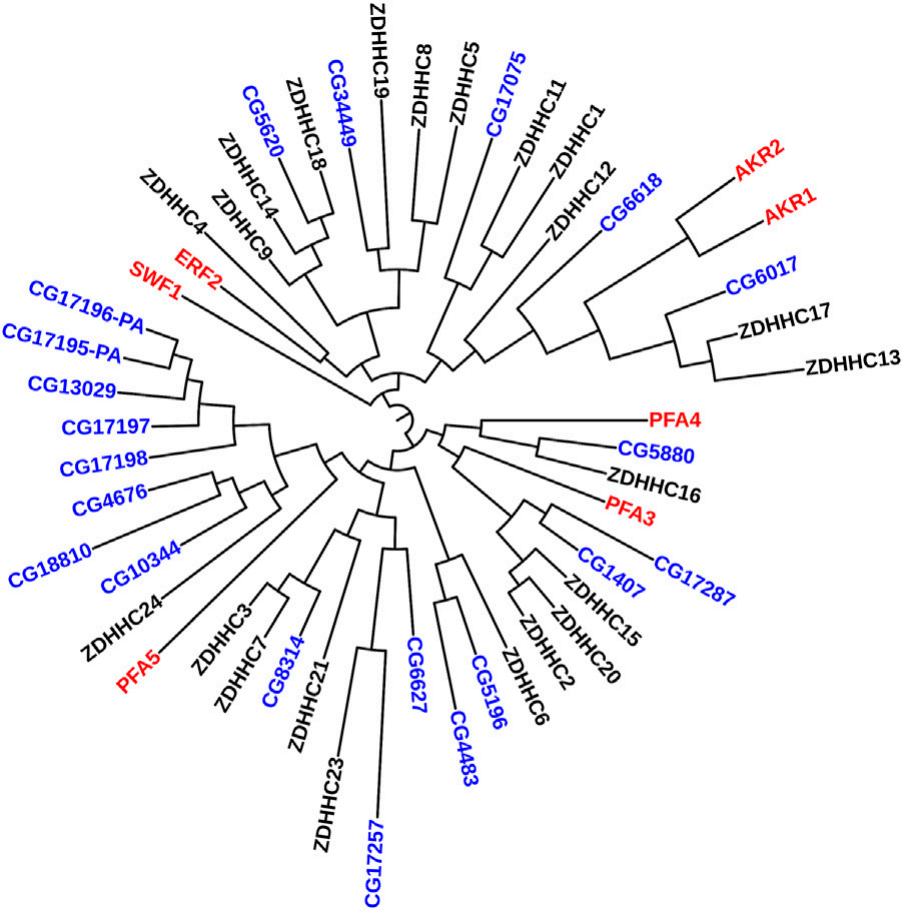
Phylogenetic Tree comparing PATs in yeast, flies, and humans. Maximum Likelihood midpoint-rooted phylogenetic tree of full-length ZDHHC proteins from *Saccharomyces cerevisiae* (red), *Drosophila melanogaster* (blue), and Homo sapiens (black) generated in Mega 11 software with 100 bootstrap replication and Jones-Taylor-Thornton model for amino acid substitution model (Tamura et al., 2021). The *Drosophila* PATs that cluster together (CG17196-PA--CG10344) are expressed only in fly testes.

Similar results have been recapitulated in flies. Presynaptic localization for dHIP14 (fruit fly orthologue for ZDHHC17) (CG6017 in Figure 2) has been reported in the central nervous system as well as the neuromuscular junction (Ohyama et al., 2007; Stowers and Isacoff, 2007). There, dHIP14 is involved in subcellular distribution of a variety of neuronal proteins in flies, including the synaptosomal associated protein of 25 kDa (SNAP-25) and cysteine string protein (CSP), and is required for photoreceptor synaptic transmission, thus suggesting palmitoylation as a possible mechanism to rapidly regulate synaptic efficiency. Indeed, dHIP14 was shown to be required for synaptic vesicle exocytosis and loss of dHIP14 led to mislocalization of SNAP25 and CSP. Moreover, dHIP14 mutants show exocytic defects at low-frequency stimulation and a nearly complete loss of synaptic transmission at higher temperatures, demonstrating the importance of dHIP14 in neurotransmitter release (Ohyama et al., 2007). This suggests that ZDHHC17’s neuronal functions are conserved across species.

##### 3.3.2 Alzheimer disease (ZDHHC12, ZDHHC7, and ZDHHC21)

Alzheimer Disease (AD) is a progressive neurodegenerative disorder resulting in brain atrophy and increased cell death, characterized by plaque formation within the brain, primarily consisting of the amyloid-beta (Aβ) protein. Aβ is formed by the sequential cleavage of amyloid precursor protein (APP) by β- and ɣ-secretases. Thus far, nearly all the primary players in AD have been shown to be palmitoylated, including APP (Bhattacharyya, Barren, and Kovacs, 2013), two ɣ-secretase subunits, APH1aL and Nicastrin (Meckler et al., 2010), and β-secretase 1 (BACE1) (Benjannet et al., 2001).

AD is the most common form of progressive dementia characterized by the accumulation of Aβ or tau protein aggregates (Nah, Yuan, and Jung, 2015; W; Zhang et al., 2022). The Aβ peptides are generated as a cleavage product of the APP. APP palmitoylation at C186 and C187 by ZDHHC7 and ZDHHC21 is required for APP to exit the ER and its normal processing (Bhattacharyya, Barren, and Kovacs, 2013). Increased palmitoylation of APP improves targeting of APP to lipid rafts where APP is cleaved, forming increased levels of Aβ aggregates. While treatment with palmitoylation inhibitors reduced the presence of palmitoylated APP in lipid rafts as well as the Aβ aggregate production, thus suggesting a role of palmitoylation in AD disease pathogenesis (Bhattacharyya et al., 2016). APP is cleaved by BACE1, followed by subsequent cleavage by γ-secretase to produce Aβ fragments. BACE1 is palmitoylated at four sites; C478, C482, and C485 in the cytosolic region (Benjannet et al., 2001; Vetrivel et al., 2009; Motoki et al., 2012), and C474 in the transmembrane domain (Vetrivel et al., 2009; Motoki et al., 2012). Studies have reported contrasting effects of BACE1 palmitoylation on APP processing. Two separate studies by the Thinakaran and the Araki groups demonstrated that mutation of the four cysteines to alanine resulted in reduced lipid raft localization in heterologous cell lines and primary neuronal cultures (Vetrivel et al., 2009; Motoki et al., 2012). However, no loss in BACE1 protein stability or subcellular localization was observed (Vetrivel et al., 2009). The non-palmitoylated form of BACE1 did not affect the APP processing and Aβ production in comparison to the wild-type BACE1 despite the loss in lipid raft association. Thus, indicating that β-cleavage of APP by BACE1 is not influenced by lipid raft microdomains (Vetrivel et al., 2009; Motoki et al., 2012). Conversely, in 2011, the Thinakaran group demonstrated that BACE1 engineered to be localized onto the lipid rafts by the addition of glycosylphosphatidylinositol (GPI) led to improved Aβ production (Vetrivel et al., 2011). Since localization to the lipid raft was palmitoylation dependent, this indicates that BACE1 palmitoylation may be contributing to APP processing. An *in vivo* study conducted in a knock-in BACE1-palmitoylation deficient mouse model revealed that although lack of palmitoylation did not affect APP processing, the synaptic activity-dependent release of Aβ aggregates increased in BACE1 palmitoylation deficient mice compared to wild-type controls as determined by the analysis of brain interstitial fluid (Andrew et al., 2017). They further showed that lack of BACE1 palmitoylation reduced cerebral amyloid burden indicating that palmitoylation may be an important modulator of amyloid burden in AD (Andrew et al., 2017).

Lipid raft-associating subunits of the γ-secretase, Nicastrin and APH-1 are also known to be palmitoylated. Nicastrin is palmitoylated at transmembrane C689 while APH-1 is palmitoylated at cytosolic C182 and C245 sites (Cheng et al., 2009). Although palmitoylation defective forms of these proteins do not affect γ-secretase assembly and function, studies have shown that the palmitoylation of these proteins is necessary for their nascent stability as well as association with lipid rafts, suggesting a role of palmitoylation in protein stability and lipid raft localization (Cheng et al., 2009; Meckler et al., 2010). Transgenic AD mice co-expressing palmitoylation deficient forms of APH1aL, an isoform of APH-1 and Nicastrin had significantly reduced Aβ aggregate deposition in the frontal cortex compared to the ones expressing WT forms of the subunits (Meckler et al., 2010). Thus, suggesting that the palmitoylation status of these proteins can influence disease pathology in AD. ZDHHC12 is implicated in regulating palmitoylation of key proteins in AD. ZDHHC12 was initially found to inhibit APP metabolism and Aβ production in AD in mouse-derived neuroblastoma cells (N2A cells) (Mizumaru et al., 2009). In addition, a recent genome-wide association study found a significant association of ZDHHC12 in structural brain connectivity in a comparison of control patients, patients with mild cognitive impairment, and patients with AD, indicating a role of ZDHHC12 in alterations of AD brain segregation and integration (Elsheikh et al., 2020). Together with data from *in vitro* studies, this suggests ZDHHC12 may be involved in the early development of Aβ plaques in AD.

### 3.4 Depalmitoylation in neurodegenerative disease

In contrast to the PAT ZDHHC enzymes, Palmitoyl-protein thioesterase (PPT) and ABHD enzymes depalmitoylate proteins. Depalmitoylation and some of the enzymes that facilitate it have been implicated in neural diseases as well as neuronal functions.

#### 3.4.1 Neuronal ceroid lipofuscinosis type 1 (PPT1)

Neuronal ceroid lipofuscinosis type 1 (CLN1), also known as infantile neuronal ceroid lipofuscinosis (INCL), is a rare (1 in 100,000 births), but one of the most lethal, inherited neurodegenerative lysosomal storage disorders caused by mutations in the *PPT1* gene resulting in PPT1 deficiency. PPT1 depalmitoylates proteins in the lysosome to facilitate protein degradation. Consequently, loss of PPT1 activity is toxic, resulting in the accumulation of pathological sphingolipids in patient cells and subsequent lysosomal enlargement (Tyynelä et al., 1993; Ahtiainen et al., 2006). Patients with CLN1 generally develop symptoms around 18 months of age, including visual defects, blindness, motor and cognitive deficits, seizures and death between 8–13 years of age (Santavuori, 1988).

Ppt1 function is well conserved from flies to humans; thus, the INCL pathologies may be due, in part, to the accumulation of various embryonic neural defects, similar to that of *Drosophila*, and may be relevant for understanding the developmental origin of neural deficiencies in INCL. Fly embryos bearing loss-of-function *Ppt1* mutations display numerous neural defects ranging from abnormal cell fate specification, missing and disorganized neurons, faulty motoneuronal axon trajectory, and mild to severe defects in the longitudinal axon bundles of the ventral nerve cord (Chu-LaGraff et al., 2010). Further, the two Ppt1 mutant strains with single point mutations (S77F and A179T) showed defects in the developing peripheral nervous system, specifically in the chordotonal neural cluster, with decreased number of sensory neurons and abnormal neuronal shapes with aberrant dendritic projections. Moreover, Ppt1 deficiency in adult flies leads to abnormal accumulation of lysosomal storage material, albeit in a different pattern than observed in human patient cells, and reduced lifespan (Hickey et al., 2006). A study overexpressing Ppt1 in the visual system of adult transgenic flies linked Ppt1 function to synaptic vesicle cycling, endo-lysosomal trafficking, synaptic development, and activity-dependent remodelling of the synapse (Buff, Smith, and Korey, 2007). Together, these results suggest that Ppt1 is essential for synaptic and lysosomal activity, and for proper neuronal cell fates and organization in early development.

A study of brain tissue from INCL patients and a constitutive *Ppt1* knockout (*Ppt1*-KO) mouse model linked rapid neurodegeneration to increased apoptosis indicated by elevated levels of caspases 3 and 9 and cleaved poly-ADP ribose polymerase enzyme (PARP) (Kim et al., 2006). Levels of superoxide-dismutase 2 (SOD-2) in INCL patient hippocampus and *Ppt1-*KO mouse brains were also increased indicating elevated oxidative stress and generally associated with higher reactive oxygen species (ROS). This was confirmed in cultured neurospheres from *Ppt1*-KO and wild-type mice. *Ppt1*-KO neurospheres had elevated levels of ROS and SOD-2, as well as SOD-2 activity (Kim et al., 2006). Together, these results suggest the rapid neurodegeneration in INCL patients and in *Ppt1*-KO mice is likely caused, in part, by a combination of ER-stress-mediated caspase-12 activation (mediates caspase-3 activation and apoptosis (Zhang et al., 2006)) as well as elevated ROS production (causes destabilization of calcium homeostasis activating caspases-3, -9, and apoptosis) (Kim et al., 2006). Furthermore, a study using a *Ppt1*-KO mouse model found therapeutic potential in supplementing the lack of PPT1 function by using a depalmitoylation molecule, N-tert-butyl-hydroxylamine (NtBuHA). Specifically, NtBuHA crossed the blood-brain barrier, depleted lysosomal ceroid, suppressed neuronal apoptosis, slowed neurological deterioration, and extended lifespan (Sarkar et al., 2013). As such, the deficiency of PPT1’s depalmitoylating activity is integral to the development of CLN1. Indeed, in a recent study, the same group found reduced ZDHHC5 and ZDHHC23 levels in the brains of the same *Ppt1* KO mouse model (Sadhukhan et al., 2021). Moreover, membrane-bound APT1 levels were suppressed, which stimulated microglia proliferation through increasing plasma-membrane H-Ras. In turn, this neuroinflammation phenotype was rescued by treatment with NtBuHA (Sadhukhan et al., 2021). These studies suggest that NtBuHA may be used as a therapeutic to lower palmitoylation in the brain, also indicating PPT1’s depalmitoylating function in INCL. Furthermore, altered palmitoylation of substrates of PPT1, APT1, ZDHHC5 and ZDHHC23 likely contribute to CLN1 pathogenesis. Previously, PPT1’s substrates were unknown. However, a recent study identified more than 100 novel PPT1 substrates, many of which have functions at the synapse, including channels and transporters, G-protein-associated molecules, endo/exocytic components, synaptic adhesion molecules, and mitochondrial proteins (Gorenberg et al., 2022). This indicates a role of PPT1-mediated depalmitoylation in synaptic and neuronal function and further supports PPT1’s effects on neuronal function in CLN1.

#### 3.4.2 Hereditary spastic paraplegia (ABHD16A)

ABHD16A, also known as Human Lymphocyte Antigen B-associated transcript 5 (BAT5), is a phosphatidylserine lipase recently identified as a novel protein depalmitoylating enzyme that has been linked to complex hereditary spastic paraplegia (HSP) (Lemire et al., 2021; Yahia et al., 2021; Miyake et al., 2022). ABHD16A is highly conserved in mammals, is composed of 558 residues in humans and mice, and was found to be genetically distant from other members of the ABHD family (Xu et al., 2018). ABHD16A exhibits ubiquitous expression with highest expression being in skeletal muscle and the brain (Xu et al., 2018). It is also palmitoylated (Martin and Cravatt, 2009) and localized in the plasma membranes in human platelets and mouse megakaryocytes (Senis et al., 2007).

HSP is a genetically and clinically heterogeneous group of inherited neurodegenerative diseases (Mackay-Sim, 2021). Patients with “complex” HSP display additional symptoms, including neuronal and non-neuronal features (Mackay-Sim, 2021). In one study of four patients from two Sudanese families, two *ABHD16A* null variants were identified to segregate in an autosomal recessive pattern of inheritance within these families, with a severe clinical presentation in the patients of developmental delay, intellectual impairment, spasticity, and skeletal deformities, similar to complex HSP (Yahia et al., 2021). The group further confirmed, in comparison to sibling controls, a lack of ABHD16A expression in patient fibroblasts (derived from 3 of the four patients) and found increased expression of the lipid phosphatidylserine with long-chain fatty acid, implicating ABHD16A’s role in lipid metabolism in the disease (Yahia et al., 2021). In another study of 11 patients from 6 families with diverse origins, homozygous variants of ABHD16A were identified across the *ABHD16A* gene, leading to diverse complex forms of HSP in each of the studied patients, and confirmed a lack of ABHD16A expression in fibroblasts derived from 4 patients (Lemire et al., 2021). In a third study of two affected children from a Chilean family, researchers identified a homozygous *ABHD16A* variant to lead to HSP (Miyake et al., 2022). These studies suggest various mutations within the *ABHD16A* gene may lead to diverse forms of complex HSP, but it remains unclear if this is due to ABHD16A’s role in lipid metabolism or protein depalmitoylation activity or perhaps both.

##### 3.4.3 Other links between depalmitoylation and neuronal mechanisms

While some depalmitoylating enzymes have been implicated in specific neural diseases, in other instances either depalmitoylation or depalmitoylating enzymes have been linked to neuronal mechanisms that may be involved in disease. Post-synaptic density-95 (PSD95) undergoes rapid palmitoylation cycling to regulate its functions in neurons (El-Husseini et al., 2002). Increased PSD95 levels were found to prevent the effects of Aβ on synapses by preventing Aβ′s modification of the NMDA receptor C-terminal domain (CTD) conformation, its interaction with protein phosphatase 1 (PP1), and thus preventing synaptic weakening. By treating with palmostatin B, a depalmitoylation inhibitor, PSD95 levels were upregulated and Aβ′s interaction with the NMDAR and PP1 were prevented, having a protective effect (Dore et al., 2021). Similarly, inhibiting mHTT depalmitoylation using an APT1 inhibitor in mammalian cells has a protective effect against forming aggregates (Lemarié et al., 2021). Similar to mHTT, increasing PSD95 palmitoylation may be a therapeutic avenue of research in diseases associated with Aβ, such as AD. ABHD17A, 17B, and 17C were found to selectively depalmitoylate PSD95 in rat hippocampal neurons, and may serve as potential therapeutic targets in conditions with Aβ dysfunction (Yokoi et al., 2016). APT1 and APT2 have both also been linked to regulate neuronal substrates (Milde and Coleman, 2014; Sadhukhan et al., 2021; Shen et al., 2022). Specifically, APT1 was found to depalmitoylate PSD-95 and glutamate receptors in rat hippocampal neurons, and also found to affect neuronal synaptic plasticity (Shen et al., 2022). Further, both APT1 and APT2 were found to depalmitoylate the axon survival factor NMNAT2 (Milde and Coleman, 2014).

## 4 Hitting the mark: interrelationship of palmitoylation and other PTMs

Posttranslational modifications (PTMs) govern many aspects of protein function and are strictly regulated. Therefore, it is not surprising that aberrant PTMs are intimately linked to neurodegenerative diseases (Ren et al., 2014; Didonna and Benetti, 2016). Despite in-depth studies of individual modifications in neurodegeneration, it remains a challenge to build a map of the complex interplay and regulation of all PTMs that are implicated in neurodegenerative diseases. However, it is important to recognize collective patterns of PTM combinations in different neurodegenerative diseases, referred to as the “PTM code” (Lothrop, Torres, and Fuchs, 2013). To date, a comprehensive overview of interactions in the context of palmitoylation within the PTM network has yet to be fully recorded. In this review, well-established PTMs that are involved in neurodegeneration will be of focus in examining crosstalk with palmitoylation (Calabrese, Molzahn, and Mayor, 2022; Didonna and Benetti, 2016; Ren et al., 2014).

### 4.1 Phosphorylation

The interaction of palmitoylation with phosphorylation has been studied to a greater extent than other PTMs in neurodegeneration. The combinatorial interplay of the two has an important role in protein trafficking and localization due to the reversibility of both modifications that can be rapidly and dynamically regulated (Stram and Payne, 2016; Zaballa and van der Goot, 2018). Studies have shown varied types of interactions including synergistic, reciprocal, and precursor regulation between the two PTMs (Salaun, Greaves, and Chamberlain, 2010; Shipston, 2011; Gauthier-Kemper et al., 2014; Moritz et al., 2015; Berg et al., 2016; Main and Fuller, 2022). The addition of the negatively charged phosphate group can modulate membrane binding by interacting with the positively charged head groups in the lipid bilayer or interrupt the interaction of polybasic domains of proteins with negatively charged head groups (Quatela et al., 2008; Vitrac et al., 2017). Palmitoylation also mediates membrane interactions for anchoring by increasing hydrophobicity (Zaballa and van der Goot, 2018). Therefore, it is not surprising that the two PTMs can operate synergistically. Indeed, the neuronal Growth Associated Protein 43 (GAP43), essential for axonal growth, requires both palmitoylation and phosphorylation for transport and association with the plasma membrane (Gauthier-Kemper et al., 2014). However, palmitoylation, not phosphorylation, was shown to be required for proper sorting from the soma to the tip of the growth cone, likely by facilitating active transport, as the non-palmitoylatable mutant remained diffuse in the cytosol (Gauthier-Kemper et al., 2014). Therefore, phosphorylation, in concert with palmitoylation, can regulate and promote protein trafficking and localization.

Reciprocal, or antagonistic, regulation has also been reported between phosphorylation and palmitoylation. Dopamine transporters are mutually regulated by protein kinase C (PKC)-mediated phosphorylation and palmitoylation where an increase in phosphorylation decreases palmitoylation, consequently down-regulating dopamine transport capacity, and *vice versa* (Moritz et al., 2015). This may contribute to the reduction in dopamine levels in Parkinson Disease (PD) (Bu, Farrer, and Khoshbouei, 2021). Similarly, synapsin 1, involved in regulating clustering of synaptic vesicles, is also antagonistically controlled where an increase in phosphorylation negatively regulates its palmitoylation, which is required for synaptic vesicle binding thereby hindering clustering at the synapse (Yan et al., 2022). This may contribute to the defects in *a*-synuclein synaptic vesicle docking observed in PD (Man et al., 2021; Zou, Tian, and Zhang, 2021).

In some cases, palmitoylation acts as a precursor for phosphorylation. For example, APT1-mediated depalmitoylation is required to precede phosphorylation within the Akt/mTOR/p70S6 signalling pathway (Berg et al., 2016). Regardless of the nature of regulation, aberrant phosphorylation is a prevalent characteristic of neurodegenerative diseases, particularly in those involving protein aggregation (Tenreiro, Eckermann, and Outeiro, 2014; Xia, Prokop, and Giasson, 2021). However, the crosstalk of palmitoylation and phosphorylation is highly variable; therefore, the relationship will need to be determined for individual proteins or diseases of interest. This suggests that palmitoylation should not be studied in isolation, but with the effects of other PTMs kept in mind.

### 4.2 Acetylation

Palmitoylation has been suggested to regulate acetylation in neural stem cell differentiation (Chen et al., 2016). Acetylation involves the transfer of an acetyl group from acetyl-CoA to lysines and is catalyzed by lysine acetyl transferases and de-acetylases, more commonly known as histone acetyl transferases (HATs) and histone de-acetylases (HDACs) (Didonna and Benetti, 2016). Disruptions in the balance between HAT/HDAC activity contribute to neurodegeneration. At the transcriptional level, histone hypo-acetylation at one or multiple genetic loci leads to lower expression and loss of function, or widespread transcriptional disruptions, respectively (Didonna and Benetti, 2016). At the protein level, several proteins in polyglutamine neurodegenerative diseases are acetylated and palmitoylated, including HTT in HD and ataxins in spinocerebellar ataxias. Here, protein acetylation was impeded by palmitoylation, *via* either inhibiting HAT or upregulating HDAC activity (Didonna and Benetti, 2016; Li et al., 2002; Sugars and Rubinsztein, 2003; Blanc et al., 2015; Yanai et al., 2006; Wei et al., 2014).

Similarly, tubulin acetylation may be regulated by palmitoylation. Tubulin is the structural subunit of microtubules that is critical for axonal structure and transport. Consequently, disruptions in tubulin have been linked to several neurodegenerative diseases (Sferra et al., 2020; Santiago-Mujika et al., 2021). Acetylated tubulin requires palmitoylation to facilitate microtubule assembly and stability in cilia and other compartments in HEK293T cells, primary astrocytes, ependymal cells, and neurons derived from inducible pluripotent stem cells (Tripathi et al., 2021). Two acetylation sites have been identified in tubulin: *a*-subunit K40 that faces the microtubule lumen and mediates binding with microtubule associated proteins and β-subunit K252 at the interface of the two subunits, which is thought to regulate dimerization and microtubule assembly (LeDizet and Piperno, 1987; Chu et al., 2011; Wloga, Joachimiak, and Fabczak, 2017). Interestingly, K252 and C354 are in close physical proximity (PDB ID: 1TUB) (Nogales, Wolf, and Downing, 1998; Madej et al., 2014; Thinon et al., 2018), which may suggest a potential regulatory interaction of palmitoylation at C354 and K252 acetylation in microtubule assembly. Thus, altered tubulin palmitoylation may lead to mislocalization or decreased microtubule formation and contribute to disease pathogenesis (Nekooki-Machida and Hagiwara, 2020). Taken together, palmitoylation regulation or interference with acetylation may be a common mechanism in neurodegeneration.

### 4.3 S-nitrosylation

S-nitrosylation involves the reversible addition of a nitric oxide (NO) group to cysteines and has a wide range of crosstalk with other PTMs (Hess and Stamler, 2012). S-nitrosylation plays an important role in modulating signal transduction pathways (Anand and Stamler, 2012; Corpas et al., 2015). S-nitrosylation increases with age and aberrant S-nitrosylation has been reported in a number of key proteins and neurotoxic pathways involved in various neurodegenerative diseases, including AD, PD, and HD (Nakamura et al., 2015). Because S-nitrosylation and palmitoylation both occur on cysteines, their potential interrelationship in regulating protein function is particularly interesting, but has not been well-characterized. Moreover, S-nitrosylation can be detected by the commonly used acyl-exchange assays. Therefore, caution must be taken when studying the two PTMs using the acyl-exchange assays. A high-throughput study using a mass spectrometric approach found changes in the crosstalk of the S-nitrosylation and palmitoylation of many synaptic proteins upon stress induction, suggesting that this interaction is important in the brain (Zaręba-Kozioł et al., 2021).

There may be direct competition at the target residue or the presence of NO may be targeting the addition of the reactive cysteine to CoA and blocking the formation of fatty acyl-CoA needed for palmitoylation entirely, leaning towards competitive regulation, especially in synaptic proteins (Salaun, Greaves, and Chamberlain, 2010; Hess and Stamler, 2012). Consistent with this notion, NO agents that induce S-nitrosylation inhibit palmitoylation of SNAP-25 and GAP43, which are required for neuronal plasticity and growth (Hess et al., 1993; Matt et al., 2019). Similarly, PSD-95 is S-nitrosylated and palmitoylated on the same cysteines, while upregulation of one inhibits the other (Ho et al., 2011). Inhibitory effects have also been reported in other proteins. For example, NO inhibits the SARS-CoV spike protein palmitoylation and reduces binding to angiotensin-converting enzyme 2 receptor on the host cell, thereby affecting infectivity (Akerström et al., 2009). However, this may not always be the case as NO has been shown to increase palmitoylation of H-Ras (Baker, Booden, and Buss, 2000).

Conversely, palmitoylation also regulates nitric oxide synthase, which is a source of NO within the cell (Iwakiri et al., 2006). Given that NO synthesis is coupled to S-nitrosylation in mammals, palmitoylation and depalmitoylation may play a direct role in global S-nitrosylation as a whole (García-Cardeña et al., 1996; Yeh et al., 1999; Navarro-Lérida et al., 2004; Anand and Stamler, 2012).

For example, S-nitrosylation regulates phosphatase with sequence homology to Phosphatase and Tensin (PTEN)/Akt signaling, and consequently, either promotes neuroprotective processes *via* inhibition of PTEN and activation of Akt, or inhibiting protective pathways by S-nitrosylation of Akt directly (Numajiri et al., 2011; Nakamura et al., 2013). In line with this notion, aberrant S-nitrosylation of sequestosome 1 (SQSTM1), or p62, hinders the clearance of built-up protein by directly inhibiting autophagy in PD (Oh et al., 2022). Correspondingly, the protein misfolding repair activity of protein disulfide-isomerase (PDI), which mediates ER stress by suppressing the buildup of aberrant proteins, is compromised by S-nitrosylation (Uehara et al., 2006; Nakamura et al., 2013). It is important to note that almost all of the aforementioned S-nitrosylated proteins in neurodegeneration are predicted to be palmitoylated, with some experimentally validated (Blanc et al., 2015). If palmitoylation, in general, inhibits S-nitrosylation, this provides another means to target pathogenic processes in neurodegeneration.

Taken together, S-nitrosylation may play a mechanistic role in disrupting palmitoylation-mediated protein trafficking, proteostasis, and function, or may function downstream of palmitoylation. The potential inhibitory regulation of palmitoylation needs to be further examined in the study of protein S-nitrosylation in neurodegeneration.

### 4.4 Ubiquitination

Crosstalk between ubiquitin and other PTMs is important in maintaining proteostasis. Ubiquitination refers to the conjugation of ubiquitin to lysine or N-terminal methionine of target substrates, including ubiquitin itself (Sadowski et al., 2012). Ubiquitin can also be removed by deubiquitinases (Komander and Rape, 2012; Schmidt et al., 2021). Although ubiquitin can signal non-degradative processes, the primary function of ubiquitin conjugation is protein targeting and tagging for degradation through the ubiquitin-proteasome system (UPS), which is often impaired in neurodegenerative diseases, thus contributing to protein aggregation (Sadowski et al., 2012; Didonna and Benetti, 2016; Watanabe, Taguchi, and Tanaka, 2020). This mechanism involves a cascade of enzymes, namely, E1 ubiquitin-activating enzyme, E2 ubiquitin-conjugating enzymes and E3 ubiquitin ligases (Komander and Rape, 2012; Schmidt et al., 2021). Ubiquitin signalling is one of the most important mechanisms in the pathogenesis of neurodegenerative diseases (Le Guerroué and Youle, 2021).

In many cases, palmitoylation has been shown to antagonize ubiquitination by increasing protein stability and preventing degradation. For example, sortilin turnover is mediated by ubiquitination which has several implications in neurodegeneration (Carlo, Nykjaer, and Willnow, 2014). Sortilin is a lysosomal sorting receptor which binds apolipoprotein and amyloid complexes in AD (Carlo et al., 2013; Carlo, Nykjaer, and Willnow, 2014). It is also involved in the delivery of progranulin to the lysosome for degradation, therefore contributing to the loss of progranulin which leads to frontotemporal dementia and TDP-43 pathology in ALS (Hu et al., 2010; Kumar-Singh, 2011; Carlo, Nykjaer, and Willnow, 2014; Gan, Khan, and Gitcho, 2015). Sortilin itself is protected from degradation by palmitoylation as it shuttles between the Golgi and endosomes (Dumaresq-Doiron, Jules, and Lefrancois, 2013). Similarly, palmitoylation protects endosomal SNARE proteins that shuttle between endosomes and Golgi in yeast by preventing ubiquitin ligase recognition (Valdez-Taubas and Pelham, 2005; Greaves and Chamberlain, 2007). In addition, voltage-gated calcium channels required for neurotransmitter release, such as Ca_v_2.2 (Saegusa et al., 2020), are also protected from degradation by palmitoylation (Page, Rothwell, and Dolphin, 2016).

On the other hand, palmitoylation plays an important role in the proper functioning of the UPS machinery. A number of E3 ubiquitin ligases themselves require palmitoylation. For example, palmitoylation of a rat E3 ubiquitin ligase enriched in the brain increases levels of overall polyubiquitination (Araki et al., 2003). In addition, E3 ubiquitin ligases, including Gp78, require palmitoylation in their canonical RING finger motifs for their enzymatic activity (Fairbank et al., 2012). Therefore, palmitoylation may protect proteins from degradation while trafficking within the cell, allowing them to carry out their functions. In contrast, aberrant palmitoylation can contribute to the ubiquitin-associated degradation or build-up of important proteins in neurodegeneration. Conversely, palmitoylation is implicated in defects within the UPS machinery, thus contributing to toxic protein build-up leading to cell death.

### 4.5 PTM network and palmitoylation

**FIGURE 3:**
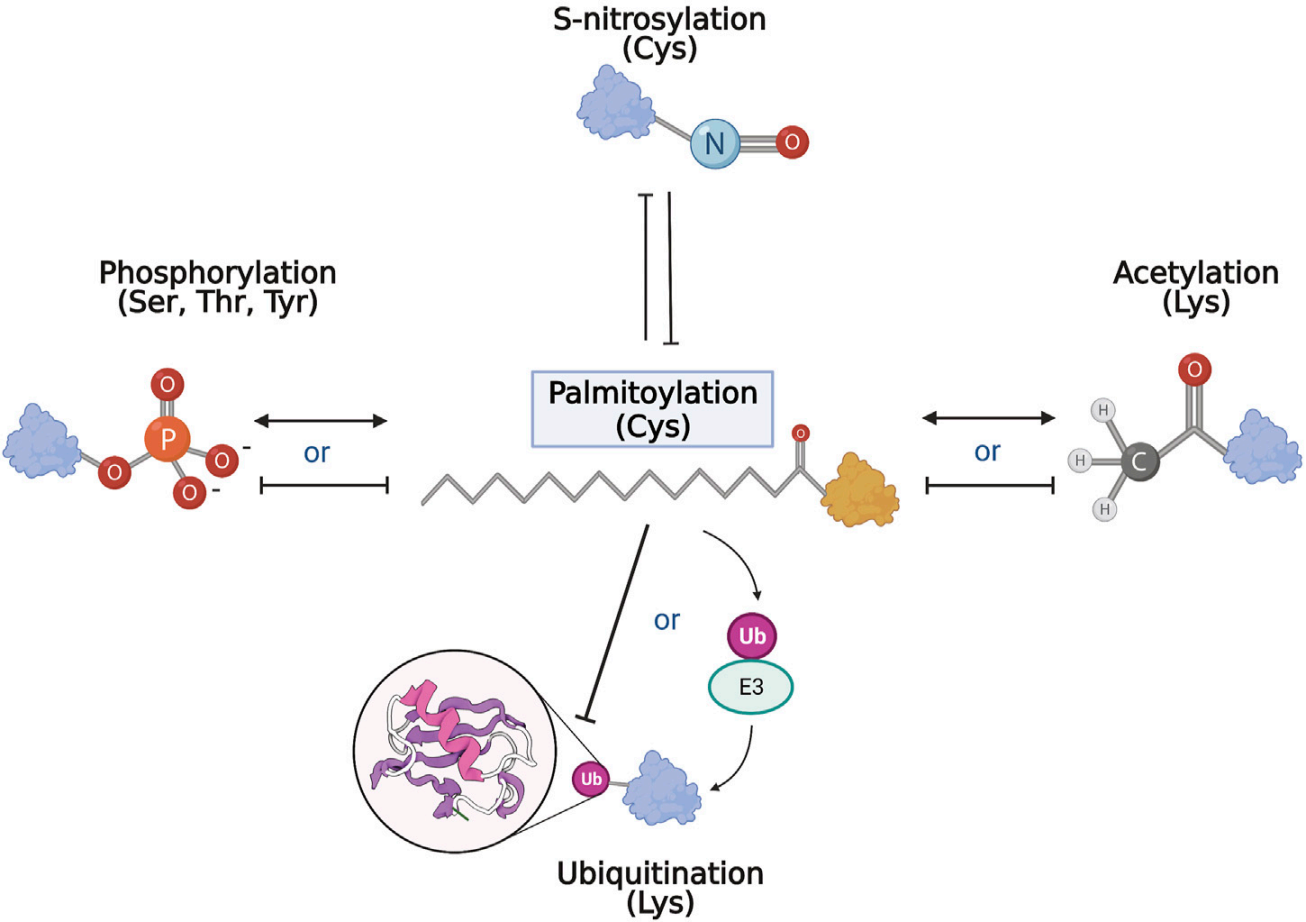
PTM network and palmitoylation. Schematic representation of the interrelationship of palmitoylation and other PTMs. Palmitoylation and S-nitrosylation mostly compete and inhibit each other on target cysteines. With phosphorylation and acetylation, both competitive and complementary interactions with palmitoylation have been recorded where palmitoylation may either inhibit or act synergistically with the other modification. Similarly, and perhaps paradoxically, palmitoylation can inhibit ubiquitination of target substrates, but is also required for activation and function of some E3 ubiquitin ligases.

Taken together, palmitoylation is closely related to many PTMs that are disrupted in neurodegenerative diseases. Although few studies examine the co-regulatory relationship of palmitoylation and another PTM on target proteins, some commonalities and themes emerged (Figure 3). So far, palmitoylation has been shown to compete with S-nitrosylation and inhibit ubiquitination directly. In contrast, palmitoylation has also shown synergy with other PTMs such as phosphorylation. Palmitoylation may precede other PTMs by facilitating correct subcellular localization of specific proteins to enable further modifications. Alternatively, palmitoylation may in turn regulate the enzymatic machinery of PTMs, such as in phosphorylation and ubiquitination, affecting the modifications on a larger scale. Regardless of the affected pathway, aberrant palmitoylation has been shown to play an important role in the pathogenesis of neurodegenerative diseases, thus presenting the need for more in-depth examinations of palmitoylation in the PTM network of neurodegeneration.

## 5 Traffic jam: palmitoylation regulates proteostasis

Following mislocalization, a primary hallmark of many neurodegenerative diseases is protein aggregation caused by deficiencies in protein turnover (Menzies et al., 2017; Ma, Attarwala, and Xie, 2019; Stavoe and Holzbaur, 2019). The cell has two primary methods to remove misfolded proteins; the UPS system, described above, and autophagy. Autophagy is an intracellular pathway responsible for the clearance of damaged organelles and toxic or aggregated proteins. The process is also important for neuronal health. With age, neuronal cells accumulate damaged organelles that can be toxic. Not surprisingly, dysfunctional autophagy has emerged as a common pathway in many neurodegenerative disorders (Nah, Yuan, and Jung, 2015).

Since neurons are terminally differentiated postmitotic cells that cannot replenish via cell division, autophagy plays an integral role in ensuring the removal of toxic cargo to maintain cellular homeostasis (reviewed in (Kulkarni and Maday, 2018; Stavoe and Holzbaur, 2019)). There are three types of autophagy pathways: microautophagy, macroautophagy, and chaperone-mediated autophagy (CMA). The best-characterized pathway is macroautophagy which will be the focus of this section, henceforth referred to as ‘autophagy’. Autophagy begins with a double membrane precursor structure called a phagophore which engulfs the target waste, consisting of damaged organelles and toxic protein aggregates (Menzies et al., 2017). Phagophores mature and close, forming a double-membraned vesicle called the autophagosome. The cargo-carrying autophagosomes fuse with lysosomes for degradation (Martin et al., 2015; Menzies et al., 2017). The autophagy pathway is regulated by several autophagy-related proteins (ATG proteins). Microtubule light chain (LC3) is an important autophagy protein involved in autophagosome formation and elongation. p62 is an autophagy receptor that delivers toxic cargo to lysosomes for degradation of the toxic aggregates (Ma, Attarwala, and Xie, 2019). p62 binds to the ubiquitinylated proteins marked for degradation and directly interacts with LC3-II. The interaction of p62 and LC3-II results in the formation of cargo carrying autophagosomes, that later fuse with lysosomes for the removal of cellular waste (Menzies et al., 2017).

### 5.1 Palmitoylation and autophagy

**FIGURE 4:**
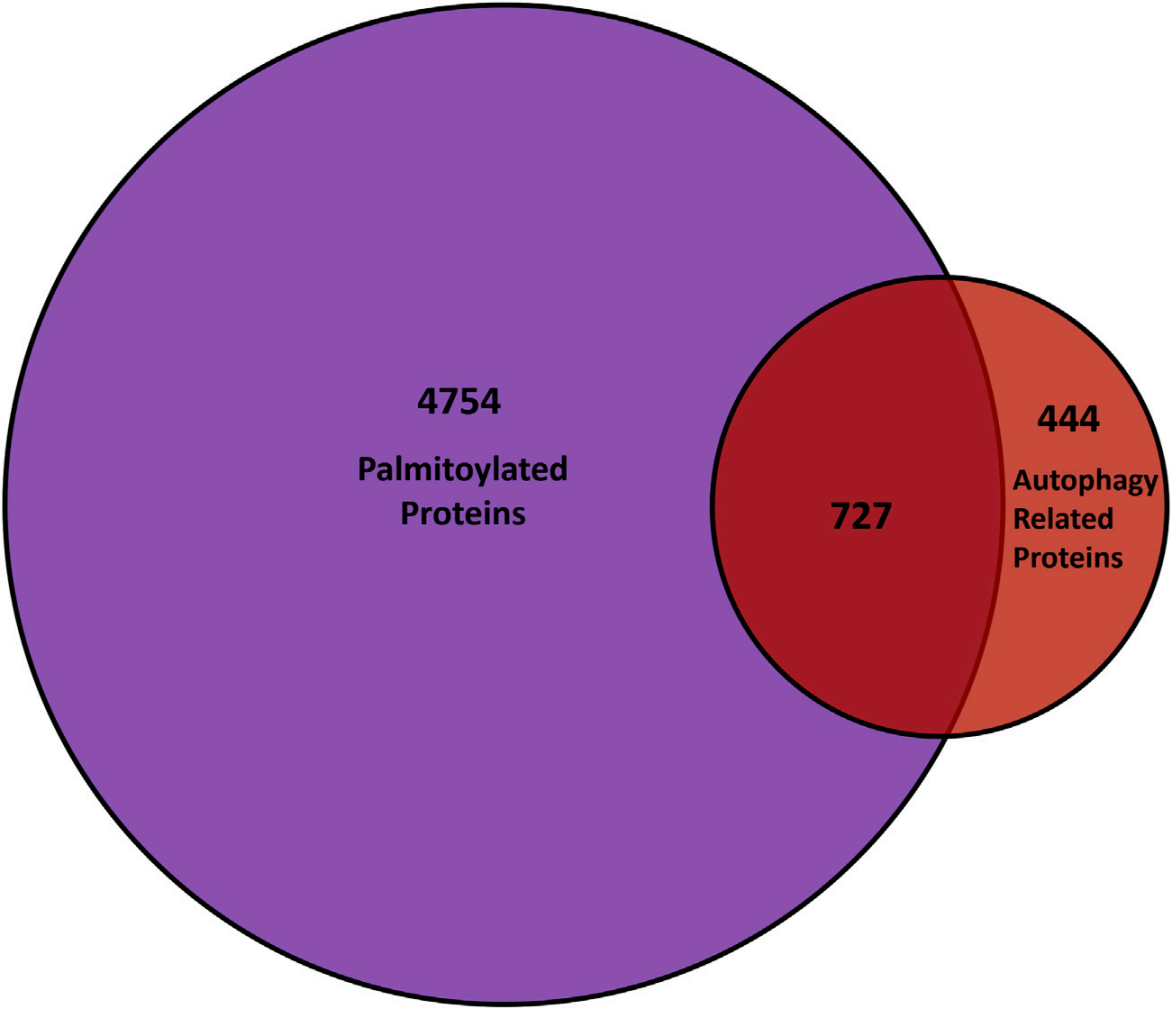
Comparison of palmitoyl-proteomes and autophagy network. The overlap between genes that encode palmitoylated proteins from the SwissPalm database (Blanc et al. 2015) and the core as well as regulatory autophagy proteins available on the Autophagy Regulatory Network (Türei et al. 2015) is represented in the Venn diagram.

Autophagy is a highly membrane-dependent process that requires proteins to quickly relocalize to membranes to initiate the formation of the autophagosome from donor membranes, inhibitory proteins need to translocate from membranes to the cytosol, and autophagosomes must fuse with lysosomes. Therefore, dynamic palmitoylation could provide a mechanism for proteins to mediate these processes. As mentioned, proteostasis deficiencies was among the top diseases enriched in palmitoylated proteins, suggesting a role for palmitoylation in autophagy (Sanders et al., 2015). In turn, several autophagy-related proteins are predicted to be palmitoylated (Ren, Jhala, and Du, 2013), while a few have recently been validated, but not in the context of autophagy. Thus, we compared the known genes of proteins from mouse, rat, and humans provided by SwissPalm (Blanc et al., 2015) and autophagy core and regulatory proteins from the autophagy regulatory network (Türei et al., 2015) (Figure 4). Remarkably, over 60% (727/1171) of autophagy regulatory genes have a proteoform that is potentially palmitoylated.

Akt is a key autophagy regulator recently shown to be palmitoylated (Blaustein et al., 2021). Akt, or protein kinase B, is a serine-threonine kinase with three isoforms Akt1, Akt2, and Akt 3. Akt has multiple targets within the cell. One of the downstream targets of the Akt pathway is the mechanistic target of rapamycin (mTOR) which is a major kinase regulator of the autophagy pathway that receives signals from various signalling pathways (reviewed in (Heras-Sandoval et al., 2014)). Activation of the AKT/mTOR pathway inhibits autophagy by mTOR activation and therefore, protects and promotes cell survival. In a recent study, Akt1 and Akt2 were reported to be palmitoylated in heterologous cells (Blaustein et al., 2021). Furthermore, they confirmed that Akt1 is palmitoylated at C344. This study revealed that palmitoylation of Akt1 not only regulates its membrane association but also showed that phosphorylation at T308, required for activity of non-palmitoylated Akt1 mutant, was downregulated significantly. Thus, suggesting that palmitoylation of Akt1 is important for Akt activity during autophagy.

Both Akt and the multimeric complex mTOR complex 1 (mTORC1) are negative regulators of autophagy and Akt suppresses autophagy through downstream activation of mTORC1. mTORC1 is an important regulator for many cellular functions, including protein synthesis, autophagy, and neuronal cell development (Deleyto-Seldas and Efeyan, 2021) and was recently shown to contain palmitoylated components, including the catalytic subunit mTOR (Sanders, Simone, and Thomas, 2019). mTORC1 suppresses autophagy activation by phosphorylation-dependent inhibition of ULK1/2, TFEB, and Atg13 in the early stages of autophagy. This prevents the expression of lysosomal and autophagy genes, thus inhibiting lysosomal biogenesis. The activation of the complex depends on the recruitment of mTOR to lysosomes, where mTORC1 assembles. In turn, recruitment of LAMTOR1 to the lysosome is also required for mTORC1 signaling and is also palmitoylated (Sanders, Simone, and Thomas, 2019). Subsequently, LAMTOR1 has been shown to interact with ZDHHC3 (Tang et al., 2022). However, investigations involving palmitoylation-specific assays are required to confirm whether ZDHHC3 is the PAT regulating palmitoylation of LAMTOR1. Results from Sanders *et al.* suggest that the activity of mTORC1 is palmitoylation-dependent in heterologous cells and hippocampal neurons (Sanders, Simone, and Thomas, 2019). Although these studies did not look at autophagy specifically or neurodegeneration, this strongly suggests that mTORC signaling during autophagy likely requires palmitoylation, further confirming the role of palmitoylation of autophagy regulators in the nervous system. Altered palmitoylation may be linked to disruptions in the PI3K/AKT/mTOR pathway in AD and PD (reviewed in (Rai et al., 2019)).

### 5.2 PATs and proteostasis

Under nutrient starvation conditions, the Golgi complex disassembles and fragments. These fragments are delivered to autophagosomes for degradation by CALCOCO1 in a selective form of autophagy referred to as Golgiphagy (Nthiga et al., 2021). CALCOCO1 is localized to the Golgi through its interaction with ZDHHC17, via the zDHHC Ankyrin-repeat binding motif (zDABM). The Johansen group identified ZDHHC17 and ZDHHC13 as interactors of CALCOCO1 (Nthiga et al., 2021), which directly binds to ZDHHC17 on the Golgi and recruits the ATG8 family of proteins at its LC3-interacting region to promote autophagy-mediated degradation of Golgi. They also observed reduced degradation of the Golgi complex in CALCOCO1 KO HeLa cell lines that were supplemented with the mutant-zDABM CALCOCO1, incapable of interacting with ZDHHC17, suggesting an important role of ZDHHC17-CALCOCO1 interaction for degradation. They predict that since palmitoylation studies have not identified CALCOCO1 as a palmitoylation substrate, the ZDHHC17 regulation of Golgiphagy is likely palmitoylation-independent. However, it will be important to confirm and investigate the effect of palmitoylation on ZDHHC17-CALCOCO1 interaction during Golgiphagy.

That said, this is a particularly interesting new avenue of research connecting autophagy and PATs in a potentially palmitoylation-independent pathway. Although ZDHHC13 and ZDHHC17 are PATs, they have several palmitoylation-independent interactors, which remain to be characterized. For instance, ZDHHC17 binds another autophagy receptor, optineurin (OPTN), in a palmitoylation-independent manner (Butland et al., 2014). It is possible that the ZDHHC17-OPTN complex may regulate another form of selective autophagy.

### 5.3 Palmitoylation beyond huntingtin in Huntington Disease

HTT is a scaffold protein that is involved in various cellular processes, including autophagy (Ochaba et al., 2014; Gelman, Rawet-Slobodkin, and Elazar, 2015; Martin et al., 2015; Rui et al., 2015; Ashkenazi et al., 2017). The build-up of toxic mutant HTT (mHTT) aggregates has been suggested to stem from impaired autophagy that is disrupted at various stages in HD. One of the characteristics of HD is the accumulation of empty autophagosomes, which is linked to disrupted cargo recognition during autophagy (Martinez-Vicente et al., 2010). HTT is now known to play multiple roles in basal autophagy (Martin et al., 2014; 2015; Ochaba et al., 2014; Rui et al., 2015; Ashkenazi et al., 2017; Ehrnhoefer et al., 2018). HTT interacts with p62 and facilitates its association with LC3 thereby playing an integral role in autophagosome formation (Rui et al., 2015). All together, these reports now implicate a role for HTT in regulating basal autophagy independent from fasting-induced autophagy. Thus, many of the defects in autophagy in HD, may be due to a loss wildtype HTT function that is also disrupted by mutant HTT.

### 5.4 Palmitoylation of HTT

Although HD is caused by a polyQ expansion at the N-terminus of HTT, there is an enrichment of palmitoylated proteins in the HD-related proteins (Sanders et al., 2015), suggesting that this PTM may be playing an important role in disease pathogenesis. HTT is palmitoylated by ZDHHC17 at the C214 site (Yanai et al., 2006; Butland et al., 2014) and depalmitoylated by APT1 and APT2 (Lin and Conibear, 2015; Lemarié et al., 2021). It was recently reported that the palmitoylation of mHTT is reduced in HD mouse models which decreases further with age (Yanai et al., 2006; Lemarié et al., 2021). Importantly, treating mHTT-expressing cells with APT inhibitors can normalize mHTT palmitoylation levels. Moreover, improved palmitoylation levels reduced mHTT aggregation and cytotoxicity in COS-7 cells and cultured YAC128 mouse neurons (Lemarié et al., 2021). A similar study demonstrated that inhibition of depalmitoylation with the APT1 inhibitor ML348 restored neuropathology in HD CAG140 knock-in mice, suggesting that overall rescue of brain palmitoylation improves HD phenotype in the CAG140 mouse models (Virlogeux et al., 2021).

The increased clearance of mHTT promoted by inhibiting depalmitoylation may be linked to autophagy by making mHTT a better substrate or redirecting mHTT to the autophagosome while also aiding wildtype HTT regulate basal autophagy. Characterizing the role of palmitoylation in autophagy regulation is important and understanding this mechanism may provide a novel therapeutic target for HD. As such, further research in this area will shed light on the link between palmitoylation and autophagy in HD. A recent study has identified several new palmitoylation sites in full-length HTT, which may provide greater insight into how mHTT is targeted for degradation (Lemarié et al., 2023).

### 5.5 Autophagy dysregulation in Alzheimer Disease

Furthermore, several studies supporting the role of autophagy dysfunction in AD pathogenesis have been reported. The first evidence emerged in 2005 when the accumulation of autophagosomes was confirmed in AD patients’ brains by immunogold fluorescence and electron microscopy techniques suggesting impairment of later stages of autophagy, particularly autophagosome to lysosome maturation (Nixon et al., 2005). This is associated with elevated p62 levels in AD patients (W. Zhang et al., 2022). It was previously reported that the purified autophagosomes from the liver of transgenic mice overexpressing APP were enriched in APP, Aβ, and proteases and secretases involved in APP cleavage (Yu et al., 2004). These findings link the build-up of Aβ aggregates with impaired autophagy. Recently, elevated p62 levels were detected in the cerebrospinal fluid (CSF) from AD and frontotemporal dementia patients, further providing evidence of autophagy dysregulation in AD (Rubino et al., 2022).

The Aβ aggregates are known to activate the mTORC1 pathway resulting in the inhibition of autophagy (Caccamo et al., 2011). Evidence so far suggests that palmitoylation plays an integral role in AD pathogenesis and mTORC1 localization and activity. Therefore, further investigations are necessary to understand the specific role of palmitoylation in autophagy regulation in AD pathogenesis.

### 5.6 Autophagy dysregulation in Parkinson Disease

A recent palmitoyl-proteome study revealed dysregulation of palmitoylation in the cortex of Parkinson Disease (PD) patients, suggesting a role of palmitoylation and protein mislocalization in PD pathophysiology (Cervilla-Martínez et al., 2022). PD is characterized by the accumulation of alpha-synuclein and ubiquitin in intracytoplasmic inclusions called Lewy bodies (Nah, Yuan, and Jung, 2015). While about 5% of PD cases are hereditary, mutations in *LRRK2* (Encoding leucine-rich repeat kinase 2, LRRK2) are considered to be one of the most common causes of genetic PD (Pang et al., 2022).

LRRK2 mutations lead to increased kinase activity (West et al., 2005) as well as the accumulation of autophagosomes (Alegre-Abarrategui et al., 2009; Bravo-San Pedro et al., 2013). Reduced co-localization of autophagosome and lysosomal markers suggests that LRRK2 mutations impair autophagosomal maturation to lysosomes (Sánchez-Danés et al., 2012). Interestingly, LRRK2 is predicted to be palmitoylated (Schapansky et al., 2014). Although they did not perform palmitoylation-specific assays, the group observed a decrease in dimerization of LRRK2 protein in total RAW264.7 cell lysates in response to treatment with 2-bromopalmitate, suggesting a potential role of palmitoylation in LRRK2 activity. Due to the broad off-target effects of 2-bromopalmitate, further investigations are necessary to characterize LRRK2 palmitoylation and its potential role in autophagy contributing to PD pathogenesis.

### 5.7 Autophagy dysregulation in ALS

Amyotrophic Lateral Sclerosis (ALS) is a fatal adult-onset progressive motor neuron disease characterized by the loss of upper (motor cortex, bulbar-onset) and lower (brainstem, spinal onset) motor neurons. About 5%–10% of ALS cases are inherited, categorized as familial ALS (fALS), while the remainder of ALS cases occur sporadically, referred to as sporadic ALS (sALS). Superoxide dismutase 1 (SOD1) was the first protein linked to ALS and mutations in the *SOD1* gene lead to 20% of fALS cases (Trojsi et al., 2020). A palmitoyl-proteomic study revealed an enrichment of palmitoylation in ALS biomarkers (Sanders et al., 2015). Indeed, palmitoylation of the fALS-linked mutant form of SOD1 (G93A-SOD1) is increased in HEK293 and motor neuron cell lines, while SOD1 palmitoylation is increased in ALS patient spinal cord tissues (Antinone et al., 2013; 2017). In cells, increased palmitoylation of G93A-SOD1 was associated with increased ER retention and decreased SOD1 maturation.

Treatment of G93A-SOD1 mice with rapamycin, an inhibitor of the mTOR pathway, leads to the accumulation of autophagosomes and yet fails to reduce levels of toxic SOD1 mutants (Zhang et al., 2011). This may be due the increased autophagosomes already detected in the spinal motor neurons of G93A-SOD1 mutant transgenic mouse models compared to age-matched controls (A. Li, Zhang, and Le, 2008) and increased autophagic flux in ALS patient lymphoblasts bearing the SOD1 mutation (Lastres-Becker et al., 2021). Since palmitoylation of SOD1 mutant is dysregulated in ALS, it will be worth investigating how palmitoylation may be regulating the autophagic flux in ALS disease.

Since the identification of SOD1’s role in ALS, over 30 genes have now been linked to ALS, many of which are required for autophagy, while the vast majority have been identified in large palmitoyl-proteomic studies (Chen et al., 2012; Cirulli et al., 2015; Blanc et al., 2015; Sanders et al., 2015). In particular, the ZDHHC17 binding partner OPTN is highly associated with ALS. This provides another strong link to palmitoylation and ALS. Furthermore, a recent genome-wide meta-analysis reported a strong association between ALS and single nucleotide polymorphisms in *ZDHHC6* (Iacoangeli et al., 2020). However, the actual role of these PATs in ALS remains unknown, therefore, it is worth investigating the role of palmitoylation on regulating ALS disease progression.

## 6 Sex differences in palmitoylation and neurodegeneration

Many neurodegenerative diseases, including AD, PD, and ALS, demonstrate sex differential etiology and diagnoses (Hanamsagar and Bilbo, 2016; Ullah et al., 2019). These sex differences are generally understudied; indeed, many therapeutic strategies are often tested in one sex over the other. As such, therapeutic strategies and a holistic understanding of disease is lacking, thus requiring an understanding of the underlying causes of sex differences. There is evidence to suggest one of these underlying causes is fatty acylation. There is a demonstrable sex difference in palmitoylation in the nervous system (Hohoff et al., 2019; Zaręba-Kozioł et al., 2021). Specifically, in a mouse model of ZDHHC7 knockout, there is a sex difference in hippocampal structure and synaptic transmission in the medial prefrontal cortex (Hohoff et al., 2019). Additionally, ZDHHC7 KO reduced anxiety-related behaviours in female mice, with no effect in males (Hohoff et al., 2019). A follow-up study compared synaptoneurosomes from male and female ZDHHC7 knockout mice for palmitoylation using mass spectrometry. Here, they found sex specifically regulated substrates of ZDHHC7 and their resulting palmitoylation, suggesting that the observed sex differences may arise from divergent substrate specificity of ZDHHC7 in male and female synapses (Zaręba-Kozioł et al., 2021).

Palmitoylation plays an important role in regulating sex hormone activity, particularly through regulating sex steroid receptors, presenting another underlying mechanism for palmitoylation to act on sex differences (Pedram et al., 2007; 2012; Cohen et al., 2021). Sex steroid receptors are transcription factors that work by activating or inhibiting gene expression, with secondary activity on rapid cellular mechanisms through membrane-bound receptor subtypes (Michels and Hoppe, 2008). Notably, both the androgen and estrogen receptors undergo S-palmitoylation and N-myristoylation (Acconcia et al., 2004; Marino and Ascenzi, 2006; Marino, Ascenzi, and Acconcia, 2006; Pedram et al., 2007; La Rosa et al., 2012). Myristoylation is similar to palmitoylation, but involves the irreversible co-translational addition of the 14 carbon fatty acid myristate to N-terminal glycines (Martin, Beauchamp, and Berthiaume, 2011). Both ZDHHC7 and ZDHHC21 palmitoylate the sex receptors (Pedram et al., 2012). Functionally, palmitoylation directs the sex steroid receptors to the cell membrane, a required step for transcriptional activity (Pedram et al., 2007). At the cell membrane, sex steroid receptors can have rapid effects on cell signaling processes (Acconcia et al., 2004). Once sex steroid receptors are transported to the nuclear membrane, they can enter and bind to DNA, regulating transcription.

There is increasing evidence suggesting that palmitoylation-derived sex differences contribute to neurodegeneration. However, current evidence linking the two is limited and palmitoylation-induced sex differences need to be further explored as a source of sexually dimorphic disease phenotypes.

## 7 Conclusion

It will be important that palmitoylation continue to be studied in the context of neurodegenerative disease. Palmitoylation is particularly relevant in post-mitotic cells, such as neurons, due to its reversibility. This reversibility makes palmitoylation a prime candidate for treatment in neurodegenerative diseases. Importantly, palmitoylation can impact cellular functions and phenotypes in neurodegeneration through several pathways, particularly protein turnover and autophagy. Autophagy is dysregulated in many neurodegenerative diseases, and many proteins involved are predicted or confirmed to be palmitoylated. Palmitoylation also interacts with other posttranslational modifications, which is another potential target in neurodegeneration. The enzymes regulating palmitoylation and depalmitoylation are also potential targets in specific diseases. For example, DLK is required for the retrograde prodegenerative signal along axons after injury in a palmitoylation-dependent manner. As highlighted above, DLK is palmitoylated by ZDHHC17. Furthermore, inhibiting DLK kinase activity has emerged as a target in multiple neurodegenerative diseases and spinal cord injury and kinase inhibitors have moved into clinical trials. Recent work from the Thomas Group has shown that blocking DLK palmitoylation is as effective as inhibiting kinase activity itself (Holland et al., 2016), and have designed high throughput drug screens to identify novel small molecules that target DLK palmitoylation (Martin et al., 2019). Indeed, targeting kinase activity tends to lead to off-target inhibition of closely related kinases but targeting DLK palmitoylation may result in more specific inhibition. Thus, understanding the molecular mechanisms of palmitoylation in neurons is likely to lead to new therapeutics or targets in neurodegenerative diseases. Ravikumar et al., 2004, Tamura et al., 2021.

As the field of palmitoylation grows and we begin to match more palmitoylation substrates with their ZDHHCs, and better understand how the interrelationship of palmitoylation and other PTMs contribute to the pathogenesis in neurodegeneration, we will be able to fully identify novel therapeutic targets.
